# Metaphase Spindle Assembly

**DOI:** 10.3390/biology6010008

**Published:** 2017-02-03

**Authors:** Tarun M. Kapoor

**Affiliations:** Laboratory of Chemistry and Cell Biology, the Rockefeller University, New York, NY 10065, USA; kapoor@mail.rockefeller.edu

**Keywords:** mitosis, cell division, microtubules, kinesin, dynein, chromosome, metaphase

## Abstract

A microtubule-based bipolar spindle is required for error-free chromosome segregation during cell division. In this review I discuss the molecular mechanisms required for the assembly of this dynamic micrometer-scale structure in animal cells.

## 1. Introduction

The equal partitioning of replicated genomes into two daughter cells depends on the assembly and function of a microtubule-based bipolar spindle, which can be several micrometers in size. The assembly of this cellular structure involves multiple steps, including the breakdown of the nuclear envelope, separation of centrosomes, organization of microtubules into a bipolar spindle, and attachment of sister chromatids to microtubules from opposite spindle poles. Many of these steps can be completed within minutes and may occur in parallel. The need for accurate chromosome segregation is likely balanced against the requirement for the rapid completion of the process as many cellular functions—including those that safeguard against damage of the genome—are largely suppressed during cell division [1].

The idea that the assembly dynamics of filaments plays a key role in spindle function came from the rapid and reversible responses to perturbation [2]. Following the identification of tubulin and the characterization of its very rapid polymerization and depolymerization rates (which are on the order of 10–50 μm/min), it became clear that tubulin’s properties are crucial for the fast timescales of spindle assembly and chromosome segregation (reviewed in [3]). Additional studies analyzing signal recovery after photo-bleaching of fluorescent tubulin incorporated into spindles revealed rapid turnover (t_1/2_: ~20 s) of tubulin at steady state [4,5]. In the following years, other imaging methods—including photoactivation of fluorescence and fluorescent speckle microscopy—confirmed these fast dynamics of tubulin and also showed that microtubules flux poleward in many animal spindles, a persistent motion at ~1–2 μm/min of the microtubule lattice towards each spindle pole [6,7]. These complex dynamics are likely to be the convolution of microtubule nucleation, directional transport, and dynamic instability, which involves the co-existence of growing or shrinking filaments and stochastic transitions between these two states [8].

**Figure 1:**
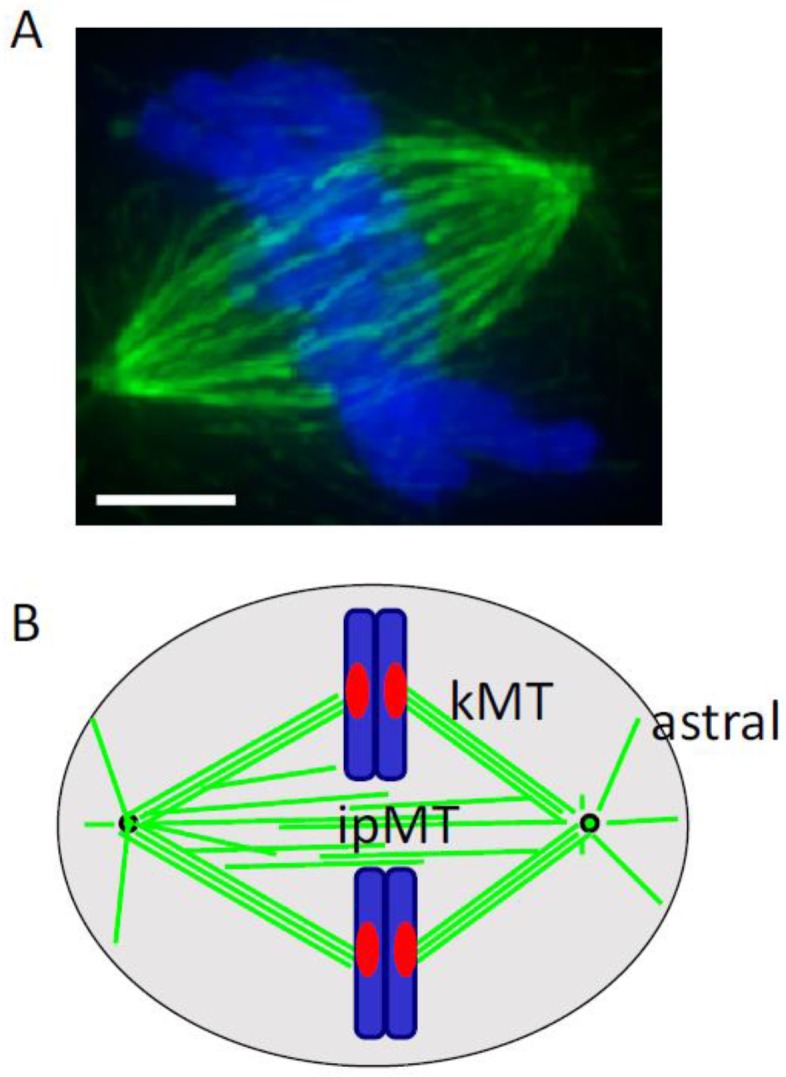
The metaphase spindle. (**A**) Overlay shows tubulin (green) and DNA (blue) in a mammalian cell. The cell was fixed and processed for immunofluorescence. Scale bar, 5 μm; (**B**) Schematic highlights kinetochore (kMT), interpolar (ipMT), and astral microtubules. DNA: blue; kinetochore: red; tubulin: green; centrosome: black circle.

At the start of cell division, the disruption of the interphase microtubule array can be abrupt, and is associated with an increase in microtubule polymer levels, as well as an increase in turnover [9]. The interphase microtubule array is typically arranged around one organizing center. In dividing cells, the separating centrosomes help organize two interacting filament arrays. Direct measurement of the dynamics of individual filaments has revealed the specific changes in dynamic instability parameters—including an increase in catastrophe frequency and the duration of depolymerization events—as cells enter mitosis [10]. A recent study which tracked microtubule dynamics in 3-D showed that the filament growth velocities in metaphase can be twice the interphase growth rates [11]. Together, centrosome separation and these changes in tubulin polymerization dynamics help to assemble the bipolar spindle, which is comprised of three types of microtubules (Figure 1). First, kinetochore microtubules link kinetochores to spindle poles, and directly contribute to chromosome motion. Second, interpolar microtubules interact with microtubules from the opposite spindle pole, but do not directly interact with kinetochores. These filaments help establish the spindle’s shape and mechanical framework. Third, astral microtubules, which extend from each spindle pole to the cell cortex, help orient and position the cell division apparatus.

The persistent and fast turnover of spindle microtubules suggests that this micrometer-scale structure continuously rebuilds itself. The spindle not only maintains its shape, but can correct defects. For example, when a microneedle is used to “cut” the spindle, it recovers its bipolar shape [12]. What is even more remarkable is that when two spindles are close enough to interact, they can fuse to form a single spindle of the same size as the original individual spindles [13,14,15]. One might assume that spindle size and shape are somehow intrinsically set by the biochemical components, and therefore such repair and fusion are possible. However, this is not likely to be the case as spindle size can change at different stages of development, when cell size reduces ~100-fold due to rapid cell divisions without cell growth [16]. Variation in the size of spindles independent of changes in the composition of the cytoplasm has been elegantly dissected in recent studies that combine the use of the *Xenopus* egg extract system and microfluidics technology [17,18]. These studies show that spindle size changes, comparable to those that occur during development, can be achieved by simply changing the volume of the cytoplasm in which the spindle assembles. Together, these findings suggest that the metaphase spindle is a dynamically self-assembling cellular structure that can autonomously maintain its organization, but can also respond to external cues.

Genome sequencing, large-scale loss-of-function studies, and proteomics have essentially identified all the proteins needed for spindle assembly in human cells. It is becoming clear that spindle assembly depends on multiple—at least partly redundant—molecular mechanisms that can act in parallel. A major challenge now is to unravel how this structure, which can be several micrometers in size, is assembled by nanometer-sized proteins. For example, we need to understand how simple geometric features, which can be 1000 times the size of the proteins required for microtubule organization, are measured in dividing cells to regulate distinct functional outputs. In this review, I discuss how metaphase spindles assemble, highlighting recent findings in the context of earlier work, and focus mainly on cell division in animal cells.

## 2. The Dynamic Architecture of the Metaphase Spindle

The metaphase spindle in animal cells is comprised of thousands of microtubules, whose densities are so high that we cannot resolve individual filaments by standard light microscopy. Therefore, insights into the architecture of the animal metaphase spindle have come from careful electron microscopy studies, which have helped establish the polarity, spacing, and overlap of the different spindle microtubule subtypes [19,20,21].

These electron microscopy studies revealed that kinetochore microtubules are organized in bundles of ~25 filaments [20,22]. The minus-ends of these filaments are located close to the spindle poles (within ~1 μm of the centriole), and the plus-ends interact with kinetochores [20]. While the number of microtubules in a bundle can vary, it does not appear to be correlated with the direction of chromosome motion [22].

The interpolar microtubules have minus-ends distributed away from the spindle pole (1–2 μm) and have mean lengths of ~4.5 μm in cells with half-spindle lengths of ~5 μm, resulting in many filaments extending past the spindle mid-plane [21]. Several bundles of two to six microtubules with close spacing (~40 nm) can be observed during metaphase, and are likely to be precursors of the microtubule bundles that persist during anaphase and become part of the central spindle. Interestingly, antiparallel microtubules are more strongly associated than parallel ones [21]. These early studies also revealed that interpolar microtubule minus-ends interact with kinetochore microtubule bundles, forming a branched “fir tree”-type arrangement. Similar microtubule branching has been described in other systems, including higher plants [23]. These early studies suggest that some of the interpolar microtubules could be nucleated at sites distal to the centrosomes [21]—an idea supported by more recent findings (see below).

Light microscopy-based analyses have revealed that the dynamics of kinetochore and non-kinetochore microtubules can differ in two ways. First, the interpolar microtubule turnover rate (t_1/2_: ~20 s) is more rapid than that of kinetochore microtubules (t_1/2_: ~420 s) [4,24,25]. Second, the rate of poleward flux for kinetochore microtubules can be ~10% slower than that for interpolar microtubules [26]. The biochemical basis of these differences is poorly understood.

The fast turnover of interpolar microtubules has raised the possibility that the lengths and positions of individual microtubule filaments may not be accurately revealed by imaging methods that require sample fixation. This may be a more significant issue in cases where these non-kinetochore microtubules comprise ~95% of the total filaments, such as the large vertebrate meiotic spindles [27]. The EB (end-binding) proteins allow growing plus-ends of single filaments to be tracked in dense networks, and have served as valuable probes to analyze microtubule organization in dividing cells [28]. However, we lack reliable reporters to track single filament minus-ends in dividing cells. The recently described CAMSAP/patronin proteins only selectively label microtubule minus-ends in interphase cells, and other proteins (e.g., ASP) have only been shown to help locate the minus-ends of microtubule bundles [29,30,31]. Therefore, analyses of microtubule distributions have relied on indirect approaches, with many studies focusing on the metaphase spindle assembled in *Xenopus* egg extracts. This cell-free system is particularly well-suited for these analyses, as it allows the addition of reagents (e.g., fluorescent proteins) at selected concentrations as well as microsurgery (needle and laser-based) [32,33].

**Figure 2:**
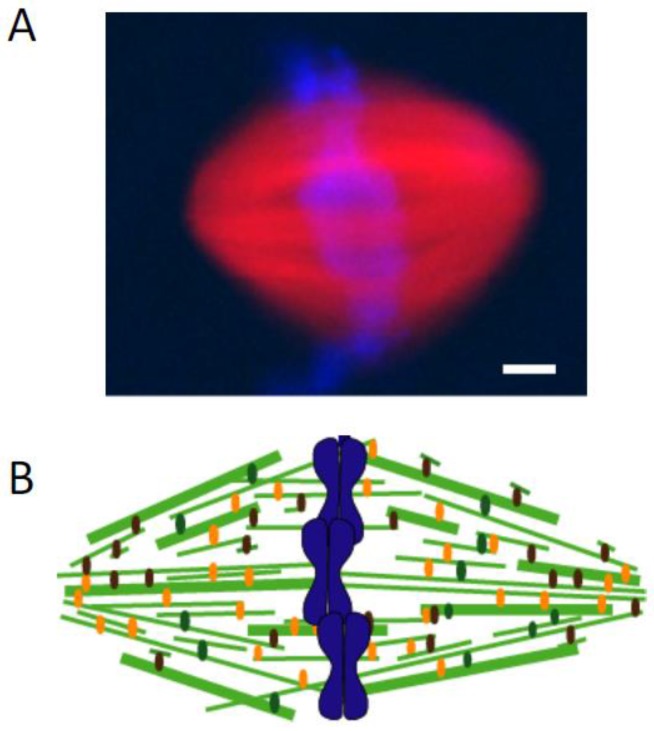
Metaphase spindle assembled in *Xenopus* egg extracts. (**A**) Overlay shows tubulin (red) and DNA (blue) in a metaphase spindle assembled around demembraned sperm DNA. Rhodamine-labeled tubulin was added to visualize microtubules, and Hoescht was used to stain DNA. Scale bar, 5 μm; (**B**) Schematic for the spindle assembled in *Xenopus* egg extracts. Tubulin: green, thicker lines indicate filament bundles; DNA: blue).

Burbank and colleagues used fluorescent speckle microscopy to determine microtubule orientation and fluorescent tubulin incorporation to localize plus-ends in metaphase spindles assembled in *Xenopus* egg extracts [34]. These data indicated that the minus-ends of microtubules are distributed throughout the spindle, with highest concentrations at spindle poles. A study from my laboratory—in collaboration with the Danuser laboratory—analyzed the motion of single fluorescent tubulin molecules in the metaphase spindle to examine microtubule organization [35]. Briefly, the poleward motion of microtubules was found to be locally heterogeneous, with standard deviations in instantaneous velocity of ~1 μm/min (~30% of the mean instantaneous velocity). Therefore, we hypothesized that the correlated motion of two single fluorophores, aligned along the spindle’s long axis, would indicate that both fluorophores likely reside on a single filament. The distance between these two fluorophores would be the minimum length of that filament. A mathematical model based on the measurements of hundreds of such fluorophore pairs indicated that the most of the non-kinetochore microtubules are shorter than the spindles’ half-length. In addition, our data indicated that these relatively short filaments are distributed throughout the spindle, consistent with a tiled organization of these microtubules in the metaphase spindle (Figure 2) [35]. Evidence for a tiled array of spindle microtubules was also obtained from elegant electron tomography and 3-D modeling studies of *Caenorhabditis elegans* oocyte meiotic spindles [36].

A more recent study combined microsurgery and quantitative fluorescence microscopy to analyze spindle microtubule length and position in metaphase spindles assembled in *Xenopus* egg extracts [37]. In this study, a laser was used to rapidly cut thin (~0.1 μm) rectangular regions perpendicular to the spindle’s long axis. The microtubule plus-ends generated by the cuts rapidly depolymerize, while the new minus-ends persist. As there is antiparallel microtubule overlap, the fluorescence intensity reduction due to filament depolymerization propagates towards each spindle pole. The relative ratios of these reduced intensity regions can be used to determine the relative orientations of the microtubules at the site of the cut. Using two laser cuts and a computational model, the authors estimated the plus- and minus-end densities and the lengths of microtubules at different locations in the metaphase spindle. These analyses revealed that microtubule lengths are exponentially distributed at all spindle locations, with mean lengths being shortest near the poles (2 μm) and longest in the middle (13.7 μm). Suppression of microtubule poleward flux removes this spatial variation in microtubule length distributions, resulting in a mean microtubule length of ~7 μm. Remarkably, this mean length is similar for microtubules nucleated in the cytoplasm, away from the spindle, by *Tetrahymena* pellicles [37], or centrosomes [38]. Based on these data and other findings, the authors propose that microtubule stability does not vary across the spindle, consistent with earlier single-fluorophore-based analyses [39]. Instead, they propose that spindle microtubule organization depends on the spatial variation of nucleation—which is highest at the center of the spindle—and directional transport-dependent sorting of microtubules [37]. Additional studies are needed to further test this model in different cellular contexts and dissect where and how microtubules are nucleated during cell division.

Together, these findings shed light on the dynamic architecture of the metaphase spindle. The more stable kinetochore microtubule bundles extend from kinetochore to spindle pole [20]. The more dynamic interpolar microtubules are likely distributed across the spindle in a tiled-array [34,35,36,37].

## 3. Micromechanics of the Metaphase Spindle

The earliest observations of cell division suggested that forces acted on chromosomes during segregation [40]. The studies by Nicklas were the first to provide a direct measurement of these forces [41]. He used force-calibrated glass microneedles to oppose the forces generated by the spindle to move chromosomes during anaphase and found that in grasshopper spermatocytes nanonewton-scale forces were needed to stall anaphase chromosome motion. Remarkably, these forces were 10,000-fold greater than what was needed to move chromosomes, not attached to the spindle, in the cytoplasm of the same cells.

Active forces which involve the conversion of chemical energy to mechanical work are generated in the spindle by motor proteins and microtubule polymerization dynamics. Individual motor proteins walking to the plus- or minus-end of the microtubules can generate forces on the order of ~5 pN [42]. Microtubule assembly and disassembly can also generate forces of comparable magnitude [43]. These active forces are balanced against each other, and against elasticity and friction, the passive forces in the bipolar spindle. Here, I focus on some recent advances in our understanding of the metaphase spindle’s micro-mechanical properties.

In principle, elasticity of the spindle can be related to that of microtubules, whose flexural rigidity has been directly measured [44]. However, establishing a precise relationship between these measurements of individual microtubules and those in the spindle requires an understanding of the number of filaments in bundles, the number and type of crosslinks (e.g., do they resist relative filament motion), and the properties of the surrounding medium. The major source of friction in the spindle is likely due to the breaking of non-covalent bonds (e.g., between microtubules and associated motor or non-motor proteins during motion). The magnitude of this resistive force increases with the rate of motion. Valuable insights in to the viscoelastic properties have been gained through analyses of cytoskeleton networks reconstituted with purified proteins [45,46,47]. However, unlike these well-studied polymer networks, the spindle is anisotropic (e.g., microtubule orientation, microtubule types, distribution of binding proteins) and the polymers are dynamic.

To directly probe the micro-mechanical properties of the metaphase spindle, my laboratory in collaboration with the Ishiwata laboratory focused on bipolar spindles assembled in *Xenopus* egg extracts [48,49,50,51,52]. There are no cell membranes in this system, and force probes can directly contact spindles that “float” in the cytoplasm and are stable for several minutes. We first employed cantilever-based probes, and found that the spindle’s response to small deformations was viscoelastic, and larger compression resulted in more plastic responses [48]. This study, along with work from other laboratories [53,54], indicates that spindles’ deformation response depends on the orientation of the applied force. In particular, we found that ~4 nN force was needed to shorten the metaphase spindle by ~1 μm along its pole-to-pole axis. Less force was needed to compress the spindle across its width [48]. These differences are likely linked to the orientation of microtubules that mainly align with the spindle’s long axis.

The cantilever-based set-up was not well-suited for fluorescence imaging, and therefore we switched to force-calibrated glass microneedle [50]. We devised a two-needle system with one stiff needle (stiffness >50 nN/μm) that could be used to apply force and another flexible needle (stiffness 0.2–0.5 nN/μm) whose bending could be used to measure force. Both needles were passivated to reduce non-specific associations with spindle components. These needles were inserted into selected sites within the spindle, and forces were applied in different orientations. This set-up also allowed us to apply forces across a wide range of timescales. This is important, as dynamics in the spindle occur across a similarly wide range of timescales, with motor proteins stepping quickly (~10–100 ms), turnover of interpolar microtubules occurring at intermediate timescales (~10 s), and kinetochore microtubule turnover being much slower (~5 min).

Our analyses revealed that the spindle’s response to deformations along the long axis is mainly viscous [50]. Based on these measurements, we can estimate that a microtubule moving at the rate of poleward flux would experience a frictional force of 10–20 pN/μm, suggesting that the active force—likely generated by a few motor proteins—would be of this magnitude.

Along the spindle’s short axis, the response to deformation is a more complex timescale-dependent combination of viscosity and elasticity [50]. The viscous response is highest on the timescale of tens of seconds. Importantly, this timescale matches that of chromosome motion and suggests that the deformation associated with the motion of a chromosome—which is large compared to the average mesh size of spindle microtubules—would be dissipated locally with limited effect of the spindle’s overall stability. The spindle’s response to deformations is more elastic at slower or faster timescales. The elastic response to short-acting forces can be linked to interpolar microtubule mechanics, while that to more persistent forces can be linked to kinetochore microtubule dynamics.

In a more recent study using force-calibrated microneedles that were not passivated (i.e., coated to block non-specific interactions), we were able to stretch spindles by applying forces at each pole [52]. The spindle’s response to these forces can be described by a Zener-type model—the model that also describes responses to forces along the spindle’s short axis [50,52]. The elastic stiffness and frictional coefficients were 5–7-fold greater along the spindle’s long axis compared to the short axis. The next major steps are to combine these measurements with biochemical perturbations to link the mechanical responses to specific protein activities and dynamics.

A tensile element that is not comprised of microtubules referred to as the “spindle matrix” has been hypothesized to play an important role in spindle assembly [55,56,57]. A variety of proteins (e.g., NuMA (Nuclear Mitotic Apparatus protein) [58], Skeletor [59], and nuclear lamins [60]), poly(ADPribose) [61], or endoplasmic reticulum (ER) membranes [62]) have been proposed to be components of such a “spindle matrix”. A recent study has also revealed interesting biophysical properties of a protein that may be associated with the spindle matrix, involving phase-transitions to form liquid droplets [63]. Based on all these studies, it appears that many of these proteins, other bio-polymers, or membranes may contribute to spindle organization in different systems. However, a direct contribution to spindle mechanics has not been firmly established. Our studies directly probing spindle mechanics [48,50], along with another study by Gatlin and colleagues [54], do not support the hypothesis that a non-microtubules-based structure in the spindle makes a substantial contribution to its overall mechanics.

## 4. Overlapping Mechanisms of Microtubule Formation

There are three major mechanisms for microtubule formation during spindle assembly.

### 4.1. Centrosomes as Sites of Microtubule Nucleation

The earliest models for spindle formation considered the centrosome—an organelle occupying a central position in the cell—to be the organizing center for microtubules [40]. The centrosome is comprised of two centrioles that organize hundreds of proteins to form the pericentriolar material, which surrounds the centrioles. Evidence for the function of centrosomes as microtubule nucleating sites came from studies with permeabilized mitotic cells and isolated centrosomes from mitotic cells [64,65,66,67]. These studies also revealed that the centrosome matures (or “ripens”) upon mitotic entry [66]. Consistent with cell cycle-dependent changes, centrosomes from mitotic cells were found to generate ~five-fold more microtubules than those isolated from interphase cells [68]. This study also showed that the capacity of the centrosome to nucleate microtubules does not depend on centriole number [68]. It is now clear that the centrosomes are the major sites of microtubule nucleation in many dividing cells (discussed in [19]).

The maturation of the centrosome involves the recruitment of additional pericentriolar material, and depends on at least two kinases: Polo-like kinase-1 and Aurora A kinase. For example, it has been proposed that Polo-like kinase-1 can help recruit pericentriolar proteins such as pericentrin via phosphorylation [69]. This kinase can also activate another kinase, NEK9, which in turn phosphorylates NEDD1 to help recruit γ-tubulin to centrosomes [70]. It is generally accepted that the main microtubule nucleator in cells is γ-tubulin, which functions with several associated proteins to form large multiprotein complexes [71]. The functions and regulation of γ-tubulin complexes are discussed in more detail below (see Section 8).

Early evidence indicated that microtubules—with plus-ends extending outwards—grow from centrosomes with spherical symmetry [72]. This process would allow the microtubule plus-ends to effectively “search and capture” kinetochores in the cytosol [72]. An interaction with the kinetochore could stabilize the microtubule and over time, lead to the polarization of the microtubule array. Direct evidence for this “search and capture” mechanism was obtained in vertebrate cells (newt lung cells) [73]. This “search and capture” model for spindle formation has strongly influenced research in the field. These studies revealed that additional—possibly redundant and overlapping—mechanisms must also contribute to bipolar spindle formation and proper chromosome attachment.

Many lines of evidence have revealed that centrosomes alone are not sufficient for spindle formation [74]. For example, experiments in unfertilized *Xenopus* oocytes showed that injection of centrosomes alone did not promote microtubule aster formation [75]. Centrosomes induced microtubule formation only when nuclei were also injected. In one key experiment, the nuclear envelope was prematurely ruptured during prophase, and spindle assembly was examined in living grasshopper cells [76]. Spindles formed rapidly under these conditions, but failed to form if the nuclei or the centrosomes were microsurgically removed. Along with additional tests, these experiments indicated that chromosomes and centrosomes are needed for spindle assembly in this system.

The importance of centrosome-independent spindle assembly is clear, as many cell types (e.g., plant cells and oocytes from several species) divide successfully without centrosomes [74]. Multiple lines of evidence from different experimental systems indicate that bipolar spindles can assemble without centrosomes in cells that normally have these organelles. For example, in *Drosophila*, functional spindles lacking astral microtubules (i.e., anastral spindles) assemble in the presence of mutations in proteins (asterless (asl) or centrosomin (cnn)) that disrupt centrosome function [77,78]. Remarkably, adult flies—albeit with some altered phenotypes (e.g., male sterility)—develop in the presence of centrosomin mutations [78]. It should also be noted that studies in other animal models (such as *C. elegans*) indicate that centrosomes are needed for bipolar spindle formation [79,80].

Probably the best evidence that functional spindles can assemble in somatic cells without centrosomes was obtained from two different microsurgery-based experiments. In one study, a laser was used to ablate the centrosome at the start of mitosis [81]. Bipolar spindles assembled with normal morphologies and recruited spindle pole proteins (e.g., NuMA), but not centrosome-associated proteins (e.g., γ-tubulin or pericentrin). Importantly, the kinetics of spindle assembly were similar to that in cells with centrosomes. In another study, microneedles were used to cut an interphase cell to generate a fragment that contained the nucleus, but lacked centrosomes [82]. These cell fragments entered mitosis and assembled a morphologically-normal bipolar spindle that lacked centrosomes. An important feature of these experiments is that centrosome-independent microtubule formation was revealed without artificially raising tubulin concentration (e.g., by treating cells with a microtubule depolymerizing drug or injecting additional tubulin), which may favor pathways that may not contribute significantly at normal physiological tubulin concentrations.

### 4.2. The Roles of Chromosomes and Kinetochores in Microtubule Formation

**Figure 3:**
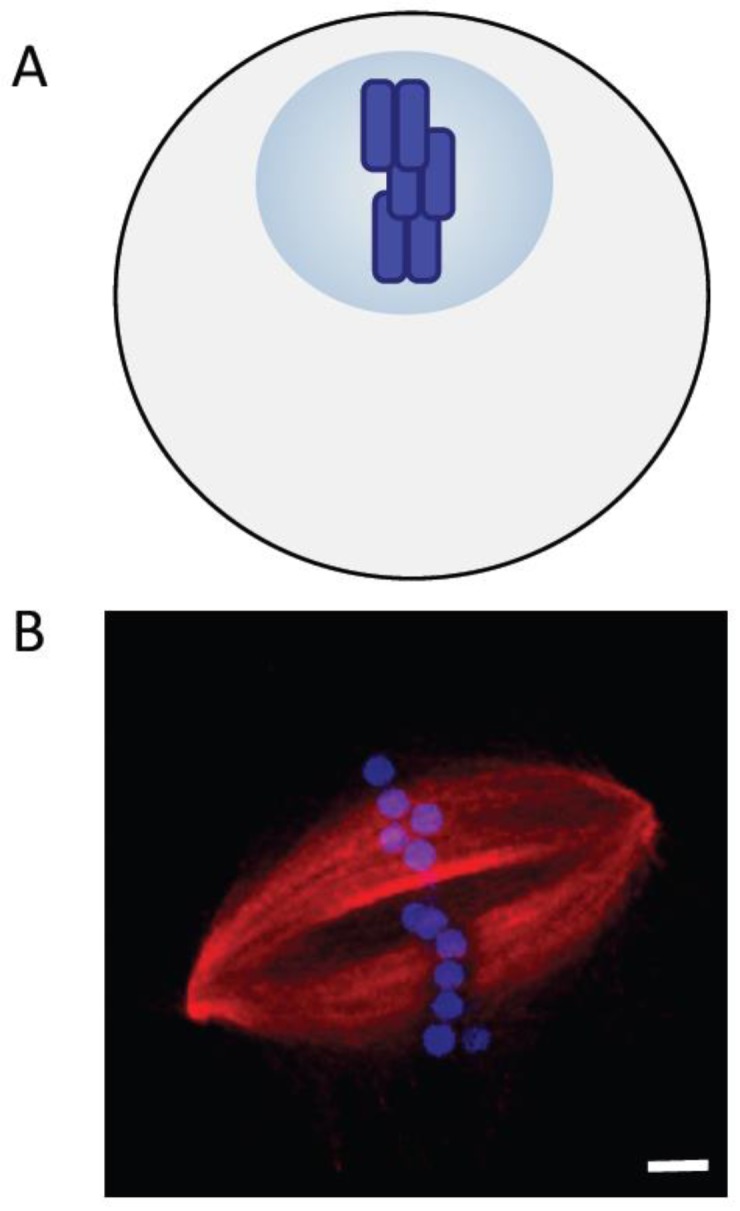
(**A**) Chromosomes (blue) generate signals to promote the formation of microtubules in their vicinity; (**B**) A spindle assembled around chromatinized DNA-beads added to *Xenopus* egg extracts. DNA: blue; tubulin: red. Scale bar, 5 μm.

Several lines of evidence suggest that the chromosomes are not merely passive cargoes, but play a key role in assembling the bipolar spindle that will eventually segregate them. Among the first direct tests of contributions of chromosomes to spindle assembly were the micromanipulation experiments reported by Marek [83]. Chromosomes in spermatocytes from two different grasshopper species were removed from the dividing cell or detached from the spindle and reintroduced at a later point. The “volume birefringence” measured using polarized light microcopy provided an estimate of the total microtubule content before and after micromanipulation. These experiments revealed that the number of microtubules in the spindle was proportional to the number of chromosomes. Nicklas and Gordon confirmed these conclusions with electron microscopy-based measurements. They found that the total length of spindle microtubules scaled with the number of chromosomes in the spindle [84]. In addition, studies of microtubule nucleation by isolated kinetochores in vitro [85] and microtubule formation in cells after recovery from treatments with chemical inhibitors of microtubule assembly [86,87] indicated that kinetochores can promote the formation of microtubules. Together, these studies also led to an important new hypothesis, that microtubule formation could be promoted by a diffusible signal generated by kinetochores (Figure 3A) [87].

A key finding was that DNA from various sources (including bacteriophage lambda) injected into an unfertilized egg and assembled into chromatin could promote local microtubule polymerization [75]. These experiments indicated that chromatin—even in the absence of a kinetochore—was sufficient to generate a signal that can promote microtubule formation. In parallel, micromanipulation studies in grasshopper spermatocytes revealed that a single chromosome can induce the formation of a mini-spindle [15]. Electron microscopy analyses indicated that only a small fraction (~4%) of the total microtubules were kinetochore-associated, leading to the proposal that the chromosome—and not just the kinetochore—contributes to microtubule formation. Evidence that chromosomes promote microtubule formation in their vicinity also came from studies in *Drosophila* oocytes [88].

The *Xenopus* egg extract system allowed additional tests of the roles of chromosomes in microtubule formation. Addition of demembraned sperm nuclei to egg extracts induced the formation of microtubule arrays that were polarized towards chromatin [89]. Compelling evidence that chromatin can induce spindle assembly in the absence of kinetochores or centrosomes came from a study using plasmid DNA attached to beads (Figure 3B) [90]. DNA-coated micrometer-sized beads were added to interphase extracts to induce chromatin formation. Upon transfer to M-phase extracts, these beads induced the formation of microtubules that self-organized into bipolar spindles within minutes.

Evidence for chromosome-dependent microtubule formation in somatic cells, in the presence of centrosomes, has come from studies in which proteins (i.e., HSET and NuMA) required for spindle pole formation were inhibited [91]. Under these conditions, centrosomes dissociated from the assembling spindle, and kinetochore microtubule bundles were still observed. These data suggest that kinetochore microtubules assemble via mechanisms independent of attachments to centrosomes in somatic cells.

Directly observing the formation of microtubules around chromosomes (or kinetochores) during mitosis is challenging due to the high density of spindle microtubules. Monastrol—a cell permeable chemical inhibitor of kinesin-5—provided a simple assay to observe chromosome- and kinetochore-associated microtubule formation in mammalian cells [92,93]. In the presence of monastrol, cells arrested with monopolar spindles as centrosome separation was inhibited. In treated cells, most chromosomes were positioned at the periphery of a radial microtubule array and oriented such that one kinetochore pointed towards the centrosome, while its sister kinetochore pointed away. Microtubules were observed forming from the kinetochore pointing away from the centrosome. As this kinetochore was shielded by chromosome arms from the dynamic centrosome-associated microtubule plus-ends, it was unlikely that these kinetochore-associated microtubules were derived from the centrosomes in the dividing cell.

There are at least two possible mechanisms for the formation of these kinetochore microtubules. First, microtubules may nucleate near the chromosome, and filament plus-ends that interact with the kinetochore get stabilized and organized into a bundle, with minus-ends pointing away from the kinetochore. Second, the kinetochore may directly nucleate microtubules such that minus-ends point away and growth occurs by the addition of tubulin at the filament plus-ends, as is the case during the polewards flux of kinetochore fibers. An elegant study in *Drosophila* S2 cells combined laser-based microsurgery and live-cell fluorescence microscopy and showed that tubulin subunits are continuously incorporated at kinetochores, even for kinetochore fibers that are severed and do not directly interact with spindle poles [94]. These data suggest that once formed, these kinetochore microtubules can grow by a poleward flux-type mechanism with minus-ends pointing away from the kinetochore.

### 4.3. Microtubule-Dependent Microtubule Formation

**Figure 4:**
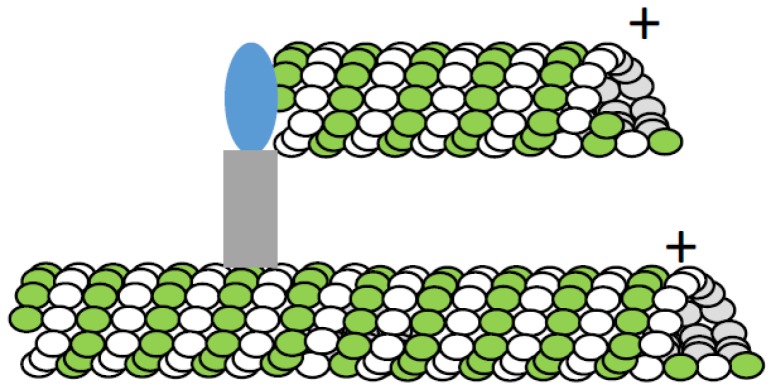
Schematic for microtubule-dependent microtubule formation. The microtubule (tubulin dimer: white, green) can recruit and activate γ-tubulin ring complex (γ-TURC, blue), possibly via the augmin complex (grey).

Studies in plants—which lack centrosomes or a readily apparent single microtubule organizing center (MTOC)—have provided valuable insights into non-centrosomal pathways of microtubule formation. An early proposal was that plants may have a “diffuse centrosome” [95]. Analyses of microtubule formation that involved tracking EB-proteins at the *Arabadopsis* cell cortex supported this hypothesis [96]. A competing hypothesis based on studies in the green alga *Nitella* suggested that microtubules could themselves recruit nucleation sites to promote the formation of new microtubules (Figure 4) [97]. In this study, microtubule formation after relief from chemical inhibitor treatments revealed highly-branched filament clusters. The microtubule-dependent microtubule formation hypothesis is also supported by analyses of γ-tubulin localization. In particular, γ-tubulin is found along microtubules within the mitotic spindle [98], and along filaments in asters assembled in vitro [99].

More direct evidence for microtubule-dependent microtubule formation came from studies analyzing MAP65 function in *Arabadopsis* [100]. In this study, microtubule formation within a microtubule bundle was directly observed. Even stronger evidence for this mechanism was obtained in an elegant study analyzing interphase microtubule organization in fission yeast [101]. Importantly, this study also provided data supporting the functional significance of recruiting γ-tubulin to the sides of existing microtubules to nucleate new ones. Additional studies tracking microtubule growth using EB-proteins in cultured insect cells suggested that a similar microtubule formation mechanism likely contributes to mitotic spindle assembly [102]. Analysis of microtubule formation in *Xenopus* egg extracts also suggested that microtubule-stimulated microtubule formation may contribute to spindle assembly [103].

There has been significant progress in our understanding of the molecular mechanisms underlying microtubule formation in dividing cells. These advances are discussed in more detail below (see Section 8 and Section 9).

## 5. The Influence of Centrosomes on Spindle Shape

The number of centrosomes in a cell is tightly controlled, and a typical vertebrate somatic cell divides with two centrosomes at opposite ends of a bipolar spindle. The importance of centrosomes in building bipolar spindles was first revealed by observations that multi-polar spindles (i.e., with more than two poles) assembled in cells with more than two centrosomes [104]. In a dividing cell with only one centrosome, bipolar spindles do not form, and a monopolar spindle—a single radial array of microtubules surrounded by chromosomes at their periphery—is observed. This has been revealed through the analysis of mutants in different model organisms, and also by specific manipulations. For example, centriole disjoining can be induced in sea urchin embryos using chemical reducing agents [105]. Under these conditions, daughter cells are generated with only one centrosome, and assemble monopolar spindles.

In many cell types, the two duplicated centrosomes separate during prophase. If this process fails, monopolar spindles can assemble. For example, when cells are treated with a chemical inhibitor of kinesin-5 (a microtubule-based motor protein), monopolar spindles accumulate [93]. Relief from chemical inhibition results in centrosome separation and bipolar spindle assembly [106]. Briefly, in addition to kinesin-5, at least three other activities can contribute to centrosome separation (recently reviewed in [107]). First, microtubule polymerization itself can generate forces to push centrosomes apart. Second, astral microtubules can interact with dynein—another microtubule-based motor protein—at the cell cortex. Dynein walking towards the minus-ends of these microtubules, or maintaining attachment to depolymerizing filament ends can pull centrosomes towards the cell cortex. Third, cortical flows generated by actomyosin at the cortex may contribute to centrosome separation via interactions with astral microtubules.

It is noteworthy that in some mutant backgrounds (e.g., *urchin,* allelic to KLP61F/kinesin-5), a bipolar spindle can form even when centrosome separation fails, indicating that bipolar spindle formation and centrosome separation can be uncoupled [108]. In this study, monoastral bipolar spindles (i.e., one pole in bipolar spindle had an associated microtubule aster while the other pole did not) were observed. More recently, a genome-wide RNAi screen revealed that mono-astral bipolar spindles can form after knockdown of a number of proteins, including the transcription factor Myb and the E2 ubiquitin conjugating enzyme UbcD [109].

Multiple mechanisms contribute to assembling bipolar spindles even when dividing cells have more than two centrosomes [110,111]. These mechanisms, which can directly cluster the extra centrosomes at two poles or act more indirectly, include the spindle assembly checkpoint, cell adhesion, and microtubule-associated proteins that help organize spindle poles (e.g., dynein, NuMA, and HSET/kinesin-14) [112,113]. It is noteworthy that multipolar spindles are not observed in the absence of centrosomes [74].

It is now generally accepted that while the centrosome may not be essential for building a functional bipolar spindle, these organelles do have important roles in dividing cells. When present, the centrosomes are the major sites of microtubule formation. In addition, centrosome-nucleated microtubules have at least two key functions. First, the centrosome-nucleated astral microtubules—which can interact with the cell cortex—play a crucial role in positioning the bipolar spindle in the dividing cell (reviewed in [114]). Second, the centrosome-associated astral microtubules can not only “search and capture” kinetochores, but also capture other microtubules. In particular, astral microtubules can interact with the minus-ends of microtubule bundles that are associated with kinetochores, but not anchored at poles [92]. This “minus-end capture” process effectively increases the kinetochore target size. In particular, the plus-ends of astral microtubules need not only find the relatively small kinetochores in the dividing cell, but can establish productive contacts with kinetochore-associated filaments that can extend micrometers beyond each kinetochore. In addition, the astral microtubules also capture interphase microtubules that remain when a cell enters mitosis. Direct imaging of green fluorescent protein (GFP)–tubulin-expressing animal cells in prophase revealed microtubules that not directly associated with the centrosome and are present at the periphery of the cell can form bundles that get transported towards the centrosome [115]. The transport of these “pre-existing” microtubules and kinetochore-associated microtubules towards centrosomes is likely mediated by the minus-end-directed motor protein cytoplasmic dynein [92,115].

## 6. The Influence of Chromosomes on Spindle Shape

Analysis of microtubule dynamics in asters formed in the presence of centrosomes and sperm nuclei added to egg extracts revealed that chromosomes polarize these filament arrays via a short-range effect on dynamic instability parameters [116]. Growth velocity and catastrophe frequencies were reduced, while rescue frequencies were increased for filaments close to or in contact with chromosomes. This study also found evidence for a weaker but longer-range effect which could guide microtubules towards chromosomes without direct interactions. For these analyses, the geometry of chromatin structures was controlled by attaching DNA to micro-patterned gold stripes on glass coverslips. A subsequent study in which chromatinized DNA-beads were used suggested that the long-range (~10 micrometer) microtubule aster polarization effect is possibly stronger than previously considered, and also induces the directional migration of arrays towards chromatin [117]. Tracking growing microtubule plus-ends in somatic cells also revealed asymmetry in microtubule growth from centrosomes [118]. During prophase and prometaphase, microtubules proximal to the nucleus/chromosomes are longer than those oriented away. Together, these studies suggest that chromosomes can influence the spatial organization of microtubule arrays that form in dividing cells.

To examine the influence of chromosomes on the spatial organization of metaphase spindles, the chromatinized DNA-bead assay has served as a powerful experimental system [90]. My laboratory, along with our collaborators, designed a setup that employed magnetic fields to align chromatinized DNA-coated paramagnetic beads into linear arrays that resembled beads on a string. These arrays ranged from ~10 to ~90 micrometers in length [119]. When these bead arrays were added to *Xenopus* egg extracts, they moved freely in the cytosol and promoted microtubule formation along their lengths. The arrays also changed shape by bending and forming kinks, likely due to interactions with microtubules that were being organized into bipolar spindles. Remarkably, the length or width of the spindles did not scale with the length of the DNA-bead string, but was similar to that of spindles that formed around unaligned DNA-bead clusters or other forms of chromatin in these extracts. In another study, Nedelec and co-workers examined the influence of chromatin on spindle assembly using chromatinized DNA-beads immobilized on surfaces with a lithographic micro-pattern [120]. Again, microtubules assembled along and proximal to the DNA-based structures. Findings from both studies indicate that the organization (e.g., aspect ratio or length) of individual spindles was largely independent of the shape of the DNA-based arrays. One key difference between the observations reported in these two studies was that multiple spindle structures were observed on the surface immobilized DNA-beads, while on the more flexible free-floating DNA bead arrays, only one spindle formed. I favor the possibility that the flexibility of the DNA-strings that my laboratory used allowed more efficient fusion of spindle poles, while in the other study, the glass surface to which the beads were fixed likely inhibited spindle pole fusion and favored spindle pole splitting. This hypothesis is supported by several observations, including the finding that when two bipolar spindles are brought close together in egg extracts, these spindles fuse to form one spindle [14].

Together, these findings indicate that while chromosomes regulate microtubule formation and polarize filament arrays, the activities of microtubule-associated proteins (e.g., motor proteins) establish spindle bipolarity and overall shape.

## 7. Dissecting the Chromosome-Based Signal for Spindle Assembly

### 7.1. Ran-GTP

Ran (Ras-like nuclear G protein) is an evolutionarily-conserved GTPase involved in diverse aspects of nuclear function. Characteristic properties of these Ras-related GTPases are that they slowly hydrolyze or exchange GTP. Nucleotide hydrolysis is promoted by GAPs (GTPase activating proteins), and nucleotide exchange depends on GEFs (guanine nucleotide exchange factors). There are advanced models for how Ran establishes the direction of nuclear transport via import and export receptor proteins during interphase [121,122,123].

The first clues that Ran regulates microtubule organization came from studies in budding yeast. A screen for genes whose overexpression suppressed the phenotype due to conditional α-tubulin mutations led to the identification of RCC1, the budding yeast homolog of RanGEF [124]. A few years later, a mutation in the budding yeast homolog of RanBP1—a protein that binds Ran-GTP and functions as an accessory factor that promotes RanGAP-mediated GTP hydrolysis—was characterized and shown to have no observable defect in nucleocytoplasmic transport, but blocked cell growth [125]. The phenotypes associated with the mutation included improper spindle positioning, likely due to failure in the formation of astral microtubules. In the same year, Nishimoto and co-workers reported the characterization of RanBPM, a Ran-binding protein [126] they had previously identified in a yeast two-hybrid screen using Ran as bait [127]. RanBPM preferentially interacted with GTP-bound Ran, associated with centrosomes in cultured cells, and its over-expression induced the formation of ectopic microtubule asters. The assembly of microtubule asters nucleated by isolated centrosomes could be suppressed by antibodies to RanBPM, and also by the addition of Ran-GTP (e.g., Ran loaded with a non-hydrolyzable GTP analog) [126]. Together, these findings indicate that Ran can regulate microtubule organization independent of its role in nucleocytoplasmic transport.

Evidence that Ran was coopted for the regulation of microtubule formation during M-phase came the following year, from studies by multiple independent research groups [128,129,130,131,132]. All of these groups used *Xenopus* egg extracts, an experimental system that allowed analyses of Ran’s contribution to microtubule organization in M-phase without concerns about its functions in other parts of the cell cycle. Reduction of Ran-GTP levels, by immunodepleting RCC1, adding mutant forms of Ran that mainly bind GDP (T24N), or adding RanBP1, suppressed the formation of microtubule asters from centrioles added to these extracts. By contrast, increasing Ran-GTP concentration via addition of RCC1 or mutant forms of Ran that are “locked” in the GTP-bound state promoted centriole-dependent aster formation. Remarkably, increasing GTP-Ran levels induced aster formation in the absence of added chromatin or centrioles. In two of these studies, the addition of GTP-locked Ran mutants (G19V bound to GTPγS [129]; or Ran L45E [131]) led to the formation of bipolar spindle-like microtubule-based structures. It was also shown that other reagents (e.g., DMSO or taxol) that promoted microtubule aster formation in these extracts did not lead to bipolar structures. Together, these findings suggest a role for Ran-GTP in microtubule formation during cell division.

The requirement of Ran’s GTP-hydrolysis cycle in regulating microtubule organization is supported by two lines of evidence. First, asters formed by Ran-GTPγS—which cannot convert to RanGDP—were smaller in size compared to those formed in the presence of RanGTP. Second, microtubule aster assembly in egg extracts depleted of the RanGEF RCC1 can be rescued by the addition of RanGTP but not RanGDP [130]. These studies, along with the fact that RCC1 is mainly bound to mitotic chromatin, led to the proposal that Ran-GTP could be the sought-after chromatin-generated “enzyme factor” that promotes spatially-restricted microtubule stabilization [87].

Ran itself does not target microtubule asters or spindles assembled in *Xenopus* egg extracts [133], suggesting that it functions through effector proteins, such as the transport receptors importin-α and β. Studies on nucleocytoplasmic transport had already established that nuclear import receptors bind their cargo in the cytosol where Ran-GTP levels are low, while in the nucleus they bind Ran-GTP and release cargoes. In particular, the transport of proteins bearing the nuclear localization signal (NLS) depends on a complex formed by importin-β that binds Ran-GTP, and importin-α, an adaptor that recognizes the NLS-bearing proteins. Importin-β can also directly recognize and transport cargoes independent of importin-α. Export receptors bind their cargoes—along with Ran-GTP—in the nucleus, and release cargoes in the cytosol after GTP hydrolysis. Guided by these models, Gruss and co-workers designed experiments to test if transport receptors contribute to Ran-dependent microtubule formation [121,123,124,125,126,127,128,129,130,131,132,133,134,135]. Specifically, they showed that the addition of importin-α, but not a mutant form that cannot bind NLS-containing cargoes, inhibited Ran-GTP-induced microtubule aster assembly in *Xenopus* egg extracts [134]. Immunodepletion of importin-α from these extracts also suppressed the formation of Ran-induced microtubule-based structures [134]. In another study, Nachury and co-workers reported that the depletion of proteins that bind a GTP-locked Ran mutant (Q69L) from egg extracts induces the formation of microtubule-based structures, while the addition of importin-β inhibits the formation of these structures [133]. Together, these findings, along with additional data, support a model in which Ran-GTP is generated proximal to chromosomes by chromosome-bound RCC1 and promotes the assembly of microtubule-based structures by locally releasing “cargoes” from transport receptors. Away from chromosomes, the concentration of Ran-GTP is lower (likely due to RanBP1 and RanGAP1 promoting GTP-hydrolysis), and the transport receptors inhibit the proteins that promote microtubule formation.

The microtubule-associated proteins TPX2 and NuMA were the first “cargoes” of the transport receptor shown to be involved in Ran-dependent microtubule formation [133,134,136]. Subsequent studies have identified several additional proteins involved in spindle assembly that can be regulated by Ran. These include non-motor MAPs (e.g., NUSAP and HURP) [137,138,139], motor proteins (e.g., XKid and HSET) [140,141], and the RNA-binding protein Rae1 [142], Cdk11 [143], and nuclear lamins [60]. Other targets, possibly via TPX2, include kinesin-5 (or Eg5/Kif11/KSP) and Aurora kinase [131,144].

Consistent with the hypothesis that Ran-GTP can function as a diffusible signal regulating microtubule organization proximal to chromosomes, a spatial Ran-GTP gradient can be detected during M-phase. The first evidence for this gradient came from studies using FRET (Forster Resonance Energy Transfer)-based sensors that have the donor and acceptor fluorescent proteins linked by a peptide [145]. One sensor was designed to detect Ran-GTP, and incorporated a peptide corresponding to RanBP1’s Ran-GTP binding region. The other sensor was engineered to detect the release of importin-α from importin-β, and employed a peptide corresponding to importin-α’s importin-β-binding domain. These sensors indicated that Ran-GTP concentration was high near chromosomes and importin-α/importin-β binding, and therefore “cargo” inhibition, were low near chromosomes in metaphase spindles assembled in *Xenopus* egg extracts.

The overall size and shape of the Ran-GTP gradients revealed by the two sensors were similar, extending over micrometers but not reaching the spindle poles, which in these spindles can be ~30 micrometers apart. Subsequent studies employed different sensors and fluorescence lifetime imaging (FLIM) rather than the measurement of donor/acceptor signal ratios alone, which can be sensitive to fluorophore concentration and bleed-through of fluorescence signal [146,147]. These measurements were consistent with a chromosome-centered Ran-GTP-dependent signal, which can release spindle assembly factors from importin-β, covering distances that extend all the way across the spindle [146,147]. A possible explanation for how this gradient could induce asymmetry in microtubule aster organization came from modeling and experimental data that indicate that the Ran-gradient may be combined with the activities of a Ran-regulated kinase (CDK11) and phosphatases [148]. FRET-based sensors also revealed the presence of a Ran-GTP gradient in somatic cells. This spatial gradient was much steeper, and extended across a shorter distance (3–4 μm) compared to what was detected in spindles assembled in *Xenopus* egg extracts [147]. A more recent study reported an even more localized spatial gradient, extending only ~2 μm in dividing somatic cells [149].

### 7.2. Chromosomal Passenger Complex (CPC)

Early ideas for how chromosomes generate a microtubule-formation signal focused on kinase- and phosphatase-based protein phosphorylation, rather than on the Ran-pathway. A specific proposal suggested that a phosphatase could be chromosomally localized, and could counteract a kinase that freely diffuses and phosphorylates microtubule-associated proteins that control filament stabilization [150]. Evidence supporting this model came from a study examining Stathmin/Op18, a 17 kDa protein that may bind tubulin subunits to suppress microtubule assembly [151,152]. Op18 can have multiple phosphorylations, many of which are present during interphase. In the presence of chromatin, additional residues in Op18 get phosphorylated. The addition of wildtype Op18 or an Op18 mutant lacking these phosphorylation sites rapidly (within ~3 min) disrupted bipolar spindles assembled in egg extracts, reducing microtubule density and overall spindle size. In egg extracts depleted of Op18, the formation of microtubules around chromatinized DNA-beads was accelerated. Other studies suggest that phosphorylation reduces Op18’s binding to tubulin [153]. Together, these data are consistent with phosphorylation suppressing Op18’s inhibitory effect on microtubule formation, and support a model in which chromosomes control the activity of microtubule assembly factors.

A FRET-based sensor whose signal changes when Op18 binds tubulin revealed a spatial gradient that is centered at chromosomes and extends towards the cell periphery [154]. These measurements and a simple calculation suggest that a phosphorylation gradient may extend 4–8 micrometers from chromosomes. However, immunodepletion of Op18—which altered early stages of spindle assembly—did not affect the shape and size of bipolar spindles assembled in egg extracts [152]. These data suggest that Op18 may not be the key effector of chromosome signals that promote microtubule assembly in dividing cells. While functional redundancy due to Op18-related proteins is difficult to exclude, the observation that a mouse knock-out of Op18 is viable is also consistent with this hypothesis [155,156].

Depletion of Polo-like kinase disrupted spindle assembly around chromatinized DNA-beads, indicating that this cell cycle kinase, which can associate with chromosomes, plays an important role in microtubule formation during M-phase [153]. Interestingly, the chromatin-induced phosphorylation of Op18 was suppressed in the absence of Polo-like kinase [153]. However, Op18 was not shown to be a direct substrate of Polo-like kinase, and subsequent studies revealed that the relevant kinase is Aurora B—a protein in the “chromosomal passenger complex” (CPC) [157,158].

Aurora B, along with Incenp, Survivin/BIR, and Dasra/Borealin form the CPC, which is enriched at the inner centromere during metaphase and associates with microtubules in the central spindle after anaphase in dividing cells [157]. CPC function is needed for multiple different aspects of cell division, including chromosome–microtubule attachment, the spindle assembly checkpoint, and cytokinesis. Compelling evidence that the CPC is needed for spindle assembly came from a study by Funabiki and co-workers [159]. Immunodepletion of the CPC via Dasra/Borealin or Incenp antibodies disrupted the assembly of spindles in *Xenopus* egg extracts. By contrast, centrosome-dependent microtubule aster formation was not affected by CPC depletion. However, these asters did not associate with chromosomes, indicating that chromosome-associated signaling was disrupted.

Kinesin-13/MCAK had been characterized as a CPC substrate whose microtubule depolymerization activity can be suppressed by phosphorylation in vitro [160,161,162]. Funabiki and co-workers showed that depletion of MCAK, along with the CPC, resulted in the rescue of microtubule formation around chromatinized DNA beads in egg extracts.

Systematic analyses of CPC function using depletion/add-back-type approaches in *Xenopus* egg extracts revealed that the Dasra subunit promoted CPC’s chromosome binding, and this interaction was needed for spindle assembly [163]. The major CPC-dependent phosphorylation site (ser-16) in Op18 was identified and used, along with a canonical CPC substrate (histone H3 ser-10), as a reporter of CPC activity in egg extracts. The phosphorylation of both substrates can be induced by the addition of chromatin to egg extracts or by the addition of stabilized microtubules [163,164]. In fact, the addition of antibodies to cluster together multiple CPC complexes resulted in kinase activation in egg extracts. This antibody-based activation could—independent of Dasra binding—promote chromosome-associated or centrosome-associated microtubules in egg extracts [163]. Follow-up studies uncovered a microtubule-binding site in the Incenp subunit, and showed that the CPC—which mainly localizes to metaphase chromosomes—can be detected within the spindle [165]. Analyses using a microtubule-targeted FRET-based sensor for CPC activity [166] suggested that the CPC can phosphorylate spindle microtubule-associated substrates [165].

Together, these data have led to a model in which the CPC, initially activated by chromosomes, must be targeted to microtubules to promote spindle formation. Near chromosomes, CPC-dependent phosphorylation likely promotes microtubule formation by suppressing the activities that increase microtubule catastrophe. Once present, microtubules can bind and activate the CPC, and thereby promote additional microtubule assembly, effectively establishing a positive feed-back loop triggered by chromosomes. This “dual detection” of chromosomes and microtubules provides a plausible explanation for how CPC-dependent microtubule formation is spatially restricted around chromosomes in egg extracts [165].

### 7.3. Interplay between Ran-GTP and the CPC

To dissect the relative contributions of the Ran- and CPC-signals to spindle formation, Funabiki and co-workers used the *Xenopus* egg extract system. In particular, they took advantage of their findings that chromatinized DNA-beads can induce microtubule assembly in egg extracts co-depleted of the CPC and kinesin-13/MCAK [159]. The addition of RanT24N, which mimics the nucleotide-free state and binds RCC1 with high affinity, suppressed microtubule formation. In addition, they showed that the addition of a GTP-locked Ran mutant to egg extracts depleted of the CPC promoted microtubule aster formation [159]. Further, the addition of RanT24N did not inhibit Op18 hyper-phosphorylation or substantially alter microtubule assembly by the antibody-mediated activation of the CPC [163]. Together, these data indicate that the CPC and the Ran pathways can act independently to promote microtubule formation in egg extracts.

The interplay between Ran and CPC-signaling was further examined by Maresca and co-workers [167]. The authors used combinations of two Ran mutants—RanT24N, which mimics the nucleotide free state, and RanQ69L, which is deficient in GTP-hydrolysis activity and mimics the GTP-bound state—to “flatten” the Ran-GTP spatial gradient during spindle assembly in egg extracts. They found that spindles did not assemble around chromatinized DNA-beads under these conditions. Importantly, mixing DNA-beads with CPC-beads (CPC linked to beads via antibodies to Incenp) promoted spindle formation, even when the Ran-GTP spatial gradient was “flattened”. Under similar conditions, the CPC-beads alone promoted microtubule formation, but not the organization of these filaments into bipolar spindles. Together, these data suggest that two independent signals are generated by chromatin to promote microtubule organization during M-phase [167].

Interestingly, the addition of EB1—a microtubule +TIP (microtubule plus-end tracking protein) that can promote microtubule formation in egg extracts—along with RanT24N also rescues spindle assembly around sperm nuclei [28,167]. As EB1 is not known to be regulated by Ran, these data suggest that Ran-GTP signals and its down-stream effectors are not required for spindle assembly when microtubule formation is at sufficiently high levels.

The contributions of both the RanGTP and the CPC pathways to chromosome-mediated spindle assembly in somatic cells is supported by a study by Wadsworth and colleagues [168]. These researchers combined the recovery of microtubule formation after treatment with nocodazole—a modification of the assay developed by De Brabander [87]—with fluorescence microscopy and inhibition of selected proteins. In this assay, the rate of microtubule formation near chromosomes was slower than that from centrosomes, but the amount of polymer generated near chromosomes was greater than that near centrosomes. Injection of importin-β suppressed chromosome-associated microtubule formation, but not centrosome-associated microtubule formation. In fact, centrosome-associated microtubule formation was slightly enhanced. These data, along with RNAi-mediated knockdown of TPX2 alone, survivin alone, or the knockdown of both survivin and kinesin-13/MCAK, indicate that the Ran-GTP and the CPC pathways contribute to the formation of microtubules proximal to chromosomes in porcine cells [168]. It is noteworthy that knockdown of kinetochore proteins did not block microtubule formation, suggesting that microtubule stabilization promoted by chromosomes does not require kinetochore–microtubule attachment [168].

Several other studies support a role for Ran-GTP in somatic cell division, including the following three. First, microinjection of importin-β’s cargo binding domain (aa 71-876) into mammalian cells (Ptk1) severely disrupted spindle assembly [133]. Second, injection of importin-β’s cargo-binding domain (aa 71-876) into other cell types (e.g., HeLa), disrupted early stages of spindle organization and caused a delay in the prometaphase-to-metaphase transition [147]. This study also showed that micro-injection of a GTP-locked Ran mutant (Q69L) led to ectopic microtubule nucleation, and aster formation and injection of full-length recombinant importin-β resulted in spindle pole “splitting” in dividing cells [147]. The authors suggest that Ran-GTP signals contribute to early stages of spindle assembly, but can be dispensable once bipolar spindles are assembled in these somatic cells. Third, studies using RNAi to knockdown Ran-GTP “effector” proteins support a role for this pathway in somatic cell division [169,170,171]. However, the findings from these knockdown studies can be more difficult to interpret, as these “effector” proteins are regulated by multiple inputs and may have functions during stages of the cell cycle other than mitosis.

The possibility that the Ran pathway has only a relatively minor role in somatic cell division is supported by different lines of evidence, including the following four. First, studies of tsBN2 cells (which lack normal RCC1 function) revealed that morphologically normal appearing spindles can assemble around chromosomes [172]. Second, siRNA-mediated knockdown of RanGAP significantly altered the shape of the Ran-GTP gradient, but did not impact bipolar spindle formation in cultured human cells [149]. Third, a study showed that in *Drosophila* S2 cells (in which a relatively steep Ran-GTP gradient can be detected during mitosis), depletion of RCC1 did not disrupt spindle assembly in the presence or absence of centrosomes [173]. Remarkably, this study also revealed that RCC1 depletion did not impact microtubule assembly during recovery from depolymerization. Fourth, a systematic dissection of the role of Ran-pathway was carried out by Khodjakov and co-workers by modifying an assay first developed by Brinkley and co-worker [174,175]. In this assay, mitosis with unreplicated genomes (MUG) is induced in cultured cells. The bulk of the chromosomes separate from small kinetochore fragments in these dividing cells. Khodjakov and co-workers used FRET-based sensors to show that the chromosomes generate spatial gradients of Ran-GTP during MUG. In addition, these chromosomes can induce asymmetry in the growth of astral microtubules, consistent with the presence of chromosome-derived signals. Remarkably, this MUG assay revealed that bipolar spindles can assemble largely independently of where the bulk of the chromosomes are positioned and where the Ran-GTP concentration is likely to be highest. The authors also showed that spindle assembly under their assay conditions did not require centrosomes, but did depend on kinetochores. Interestingly, these authors also found that inhibition of the CPC using chemical inhibitors of Aurora kinase did not suppress spindle formation during MUG.

A recent study by Needleman and co-workers may help explain these conflicting data on the role of Ran-GTP in dividing somatic cells [149]. These authors apply an approach called TIMMA (time-integrated multipoint moment analysis, a multipoint fluorescence fluctuation spectroscopy method) to determine protein concentration and measure diffusion constants at several locations in a single living cell. They find that Ran exists in fast- and slow-diffusing forms in dividing cells. The slow-diffusing form—likely bound to importins—is enriched proximal to chromosomes, while the fast-diffusing form is uniformly distributed across the cell. This method also revealed that the Ran-regulated microtubule associated protein TPX2 is also present in fast- and slow-diffusing forms. However, unlike Ran, both species of TPX2 are strongly enriched proximal to chromosomes. Microtubule depolymerization disrupts the soluble TPX2, but not the Ran, spatial gradients. These data, along with analyses of two other Ran-regulated proteins, suggest that the spatial distribution of spindle assembly factors is not only influenced by Ran, but also by interactions with microtubules. The spindle assembly factors activated near chromosomes by Ran can promote local microtubule formation, and interactions with these newly formed filaments can lead to local feed-back influencing spatial organization and function. This model can help explain how spindle size can be uncoupled from the shape of the Ran gradient. It also provides plausible explanations for findings from the MUG assays and how spindle size scale may vary with cell volume [17,18,175].

## 8. Targeting and Activating γ-Tubulin

Genetic studies of microtubule organization and function in *Aspergillus nidulans*—an important model organism in which many key mitosis genes have been discovered [176]—led to the identification of γ-tubulin as a suppressor of a conditional lethal mutation in β-tubulin [177]. Subsequent studies localized γ-tubulin to centrosomes in different cell types, and revealed that it is part of a large multi-protein complex called γ-TURC (or γ-tubulin ring complex, reviewed in [71]). It has been established that γ-tubulin has an essential role in microtubule formation in a variety of cell types, including those that do not depend on the centrosome for cell division (e.g., plants [178] and *Drophophila* oocytes [179]). Consistent with these data, γ-tubulin is also implicated in assembling microtubules from non-centrosomal sites (such as kinetochores [180]), and can be found located within the spindle [179,181]. In addition to promoting microtubule nucleation, γ-tubulin may also function as a microtubule minus-end cap [182].

Reconstituted γ-TURC complexes from *S. cerevisiae* have been characterized and found to be much less efficient in nucleating microtubules when compared to centrosomes [183]. Specific structure-guided crosslinks of the γ-TURC complex into a “closed” complex only led to modest increases in nucleation activity [71]. A possible explanation for these observations has come from a recent study by Brouhard and co-workers that shows that microtubule nucleation from templates such as γ-TURC is kinetically unfavorable in vitro [184]. They suggest that this is due to a structural mismatch between the ring-shaped templates and growing microtubule plus-ends, which may exist as sheets [185]. Brouhard and colleagues also show that microtubule-associated proteins that promote catastrophe (e.g., MCAK) inhibit nucleation and suppressors of catastrophe (e.g., TPX2, also see below) promote nucleation. These data, along with other findings, suggest different models for how microtubule-associated proteins can promote γ-tubulin-dependent microtubule nucleation. First, microtubule-associated proteins can bind and activate γ-TURC complexes. Second, an indirect mechanism would involve microtubule-associated proteins promoting nucleation by inhibiting catastrophe events to prevent the loss of newly-nucleated filaments. Third, suggested by a recent study from Surrey and co-workers (discussed in more detail below), microtubule-associated proteins can directly promote microtubule nucleation [186,187]. In this case, γ-TURC complexes may stabilize or “cap” polymers with a specific organization (e.g., a particular protofilament number).

### 8.1. Microtubule Targeting of γ-Tubulin

Clues for how γ-tubulin could be targeted to microtubules in dividing cells came from a study by Stearns and co-workers characterizing NEDD1 (or GCP-WD), a subunit of the human γ-TuRC [188]. These authors showed that NEDD1 is required for the localization of γ-tubulin at centrosomes and within mitotic spindles. Assays analyzing microtubule regrowth after drug-induced depolymerization revealed that NEDD1 is needed for centrosome-dependent and -independent microtubule formation in dividing cells. In particular, a specific phosphorylation of NEDD1 (at Ser418, likely by CDK1) helps recruit γ-tubulin to the mitotic spindle, but not the centrosome. Consistent with this localization, this mitotic NEDD1 phosphorylation was shown to be required only for centrosome-independent microtubule formation. Based on these findings, the authors proposed that NEDD1 could recruit γ-TURC to the sides of microtubules to promote microtubule-dependent microtubule formation, similar to the mechanism suggested by Tran and co-workers examining interphase microtubule organization in fission yeast [101]. They also suggest that their observations could also be explained by an indirect mechanism in which NEDD1 contributes to the proper distributions of filament minus-ends in the spindle, and γ-tubulin “caps” these filament ends. This latter hypothesis is supported by findings that inhibition of the microtubule-severing protein katanin reduces the amount of γ-tubulin in the spindle [189].

Other proteins that recruit γ-tubulin to spindle microtubules were discovered by Goshima and co-workers [109]. These authors carried out a genome-wide screen that employed high-throughput microscopy to analyze mitotic phenotypes in cultured *Drosophila* S2 cells. They first confirmed that γ-tubulin recruitment to spindle poles depends on centriolar proteins (e.g., Sas-6) and polo kinase. They found that bipolar spindles assembled after RNAi-mediated knockdown of these centriolar proteins, indicating that the recruitment of γ-tubulin to the spindle pole is not essential for cell division in these cells. They also discovered that the recruitment of γ-tubulin to the spindle depends on a set of previously uncharacterized proteins that they named Dgt2–6 (for dim γ-tubulin). The knockdown of these Dgt proteins—which also localize to spindle microtubules—reduced spindle microtubule density and caused defects in spindle organization, chromosome alignment, and cell cycle progression. Importantly, knockdown-associated phenotypes became more severe when centrosomal and Dgt proteins were co-depleted, indicating that the Dgt proteins contribute to centrosome-independent microtubule formation. Additional work revealed that Dgt proteins form a heteroctameric-protein complex that was named “augmin” [190], and is conserved across metazoans [191,192]. To recruit γ-tubulin to spindles, augmin’s Dgt6 (also named HAUS6) subunit’s C-terminal domain likely binds NEDD1 [191], the protein previously shown to be involved in recruiting γ-TURC to spindle microtubules [188].

It is tempting to speculate that augmin functions in a manner similar to the Arp2/3 complex, which can bind along the side of an actin filament and promote the nucleation of a daughter actin filament [190,193]. In such a model augmin would bind to the side of a microtubule and recruit γ-TURC to promote the formation of a daughter filament oriented parallel to the mother filament (Figure 4). Consistent with this model, an elegant electron tomography study by Kamasaki and co-workers detected a ~29 nm rod-shaped structure at microtubule minus-ends that could serve as a link to the side of another microtubule in the mitotic spindle [194]. However, it is unclear if augmin is indeed this rod-shaped structure, and additional work (e.g., immuno-electron microscopy analysis) is needed.

Currently, evidence that augmin is involved in microtubule-dependent microtubule formation comes from a study by Petry and co-workers [195]. In this study, microtubule aster formation in *Xenopus* egg extracts was induced by the addition of a GTP-locked Ran mutant (RanQ69L) and the microtubule-associated protein TPX2. The formation of branched microtubule networks could be directly observed under these conditions. Immunodepletion of augmin suppressed the organization of microtubules into asters in this assay, consistent with its role in microtubule-dependent microtubule formation. Interpreting these results is not entirely straightforward, as TPX2 itself can directly promote microtubule formation (discussed below in more detail).

The loss of augmin function has been studied in different organisms. In zebrafish, mutation in augmin leads to defects in hematopoiesis [196]. In flies, augmin mutants are viable but female sterile [197,198]. In filamentous fungus, disruption of augmin genes does not affect mitosis [199]. By contrast, the mouse knockout of the augmin subunit Dgt6/HAUS6, generated by Watanabe and co-workers, indicates that augmin is needed for mouse embryonic development [200]. Centrioles are absent for the first divisions during mouse development, and spindle assembly involves the clustering of multiple MTOCs. Interestingly, MTOC clustering fails without augmin. This phenotype is similar to what has been reported for augmin RNAi in cultured cells [192,201,202].

To dissect the role of augmin in MTOC clustering, Watanabe and co-workers overexpressed Polo-like kinase 4 (PLK4) in HeLa cells, which can lead to spindles with multiple poles [200]. Their findings using this assay—along with augmin knockdown and disruption of NEDD1-dependent γ-tubulin targeting to the spindle—suggest that the γ-tubulin associated with spindle microtubules contributes to centrosome clustering. It is noteworthy that the electron microscopy studies by Kamasaki and co-workers had found defects in centriolar microtubules after knockdown of augmin, suggesting a more direct role for augmin in centrosome organization [194]. Therefore, additional studies are needed to properly dissect how augmin contributes to centrosome (or MTOC) clustering during cell division.

In an effort to dissect augmin function, my laboratory reconstituted this hetero-octameric complex with recombinant proteins expressed in insect cells [203]. Our biochemical and electron microscopy-based studies of the “holo-complex” and different stable sub-complexes revealed how the eight proteins may interact to form a Y-shaped structure. In assays with purified proteins, augmin bound the sides of stabilized microtubules with micromolar affinity and diffused in 1-D with short association times (seconds), but did not reveal any preference for microtubule ends. It is noteworthy that these microtubule binding lifetimes are similar to what has been measured for augmin turnover in dividing cells (e.g., GFP-Dgt5/HAUS5; t_1/2_ = 4 s [190]). Further, the electron microscopy study by Kamasaki and co-workers shows that there is typically only one daughter microtubule associated with a mother filament, suggesting short-lived association of the new and the pre-existing filaments [194]. If these associations were long-lived, multiple daughter filaments would be associated with one mother filament, as daughter filaments would have nucleated additional filaments.

We also showed that the addition of augmin holo-complex and sub-complexes to *Xenopus* egg extracts promoted the formation of microtubule asters [203]. This activity is increased in the presence of RanQ69L (GTP-locked mutant) and depended on the HAUS8/Hice1-subunit’s microtubule-binding site, but did not require the domain in HAUS6/Dgt6 needed to bind NEDD1 and recruit γ-TURC. Comparisons of asters induced by octameric and sub-complexes with similar in vitro microtubule binding properties indicated that proper asymmetry and microtubule bundling in these asters required all eight subunits in the augmin complex. In these assays, we were unable to detect augmin at branch points between filaments. Consistent with our studies with purified microtubules, the augmin complexes associated along the lengths of microtubules. Therefore, additional studies are needed to determine if augmin does indeed work in a manner similar to the Arp2/3 complex, or if it promotes aster formation by directly stabilizing microtubules and promoting their bundling. These models for augmin function need not be mutually exclusive, and together may help explain augmin’s function during cell division.

### 8.2. A Direct Role for TPX2 (Targeting Protein for Xklp2) in Microtubule Formation

This microtubule associated protein was identified as a factor needed for the recruitment of the motor protein XKLP2 (human kif15, kinesin-12) to spindle poles [204]. While the potential role of TPX2 in directly regulating XKLP2 function is not yet fully understood, it is clear that TPX2 may be a protein with several distinct functions, including the spindle targeting of kinesin-5 and Aurora A kinase [205]. Here, I highlight recent progress in our understanding of its function in promoting microtubule formation and its regulation by Ran-GTP.

An important finding was that recombinant TPX2 added to *Xenopus* egg extracts can induce the formation of microtubule asters [206]. Interestingly, in another study, TPX2 was found in HeLa cell nuclear extract fractions that can promote microtubule aster formation when added to *Xenopus* egg extracts [134]. Additional analyses using assays with importin-α depleted egg extracts, GTP-locked Ran mutants, and recombinant TPX2 led to the proposal that TPX2 may be the only importin-α binding protein required for Ran-GTP-dependent microtubule formation [134]. The involvement of TPX2 and several other effectors of Ran-dependent microtubule formation has raised the question of whether it is possible that the importins can quantitatively sequester and inhibit the functions of all these proteins. This is relevant, as the concentration of NLS-containing proteins can be high, and may compete for transport receptor binding. One solution to this potential issue has been uncovered by recent structural and biochemical analyses of TPX2-importin-α binding [207]. Via its central domain, TPX2 binds at a site on importin-α that is distinct from the site bound by many NLS-containing proteins, thereby effectively reducing direct competition. However, it is noteworthy that TPX2-immunoprecipitations do not efficiently co-deplete importin-α from egg extracts, and additional analyses are needed to properly dissect this interaction in cellular contexts [134]. Importantly, additional in vitro studies have shown that purified recombinant TPX2 (albeit at high concentrations) can promote the formation of tubulin aggregates and filament bundles [208].

More convincing evidence for a direct role of TPX2 in microtubule formation and its regulation by importin-α has come from a recent study by Surrey and co-workers [186]. Characterization of full-length TPX2 revealed that it is a monomer in solution and has some preference for the GMPCPP-bound lattice and the growing tip of a microtubule, but not the end of a shrinking filament [186]. These findings, along with other data, suggest that TPX2 recognizes a specific tubulin conformation at the growing filament end, but this feature is likely distinct from that recognized by the EB proteins [209]. TPX2 also suppresses microtubule catastrophes and slows depolymerization, thereby increasing microtubule lifetimes. Surrey and co-workers also find that while TPX2 does not strongly bind soluble tubulin dimers, it can promote the formation of “stubs” that are likely multimers of tubulin. The growth of these “stubs”—which may be microtubule nucleation intermediates—is blocked by TPX2.

Human chTOG (or XMAP215/Stu2p/Dis1/Alp14 homolog) was also characterized in this study, and was shown to be a microtubule polymerase similar to other proteins in the XMAP215 family [28,186,210]. Additionally, consistent with an earlier study of other XMAP215 proteins, chTOG only weakly promoted the nucleation of microtubules. Remarkably, chTOG and TPX2 together strongly promoted microtubule nucleation [186].

Interestingly, in controlled in vitro experiments, purified importin-α/β could inhibit the nucleation of microtubules by TPX2 and chTOG, and blocked the formation of tubulin “stubs” by TPX2 [186]. These studies provide straightforward explanations for why TPX2 and XMAP215 are needed for Ran-GTP-dependent microtubule formation in *Xenopus* egg extracts, and the observation that TPX2 promotes microtubule nucleation from XMAP215 immobilized on beads [211]. Future work characterizing the TPX2-nucleated “stubs” will help strengthen this model and advance our understanding for how TPX2, along with γ-TURC and augmin, contributes to microtubule formation during cell division.

## 9. Sliding and Sorting Microtubules

The importance of the relative sliding and sorting of microtubules for spindle assembly and function was appreciated long before many of the microtubule-based motor proteins needed for cell division had been identified [212]. The identification of several kinesins required for cell division resulted from studies in the genetically-tractable fungi *Aspergillus nidulans* and *Saccharomyces cerevisiae* [176,213]. Studies in *S. cerevisiae* also provided the first compelling evidence supporting a model in which counter-acting forces generated by motor proteins help assemble spindles [213,214]. In particular, the phenotypes due to the loss of kinesin-5 (Cin8 and Kip1) function could be partially suppressed by the deletion of the kinesin-14 (Kar3) gene in *S. cerevisiae* [214]. Subsequent work from several labs in different model organisms has helped to further develop this model for how motor proteins can push or pull microtubules to assemble spindles. These mainly cell biological studies suggest how spindle length may be controlled and how microtubules may be focused at spindle poles. These findings have been extensively reviewed [53,74,107,215,216,217]. Advances in our understanding of the regulation of microtubule length—another key parameter for proper spindle assembly and motor protein-based sorting—has also been recently reviewed [210]. Here I focus mainly on recent biochemical and biophysical studies of mitotic motor proteins and how they help explain spindle assembly.

### 9.1. Kinesin-5 (or Eg5/KSP/Klp61F)

**Figure 5:**
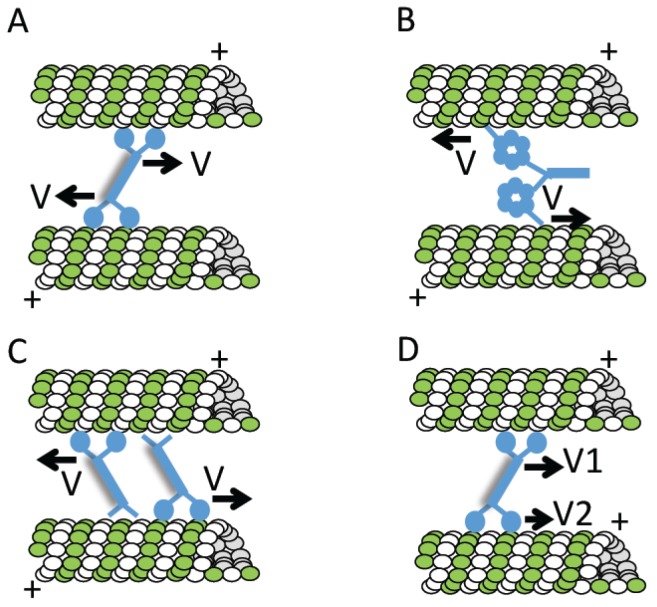
Schematics for how different motor proteins can crosslink and slide two microtubules apart. (**A**) A kinesin-5 homotetramer can walk towards the plus-end of each filament it crosslinks; (**B**) Kinesin-14 dimers crosslink microtubules via motor (circles) and non-motor (lines) domains. The motor domains can bind either filament and walk towards its minus-end; (**C**) Dynein dimers can walk towards the minus-ends of the microtubules. Each motor domain in the dimer may interact with a different filament; (**D**) Kinesin-12 homotetramers may slide parallel microtubules relative to each other by walking faster on one filament in the pair. Tubulin dimer: green, white; motor proteins: blue; V = velocity; in D, V1 is greater than V2; plus-end of the microtubule is also indicated (+).

Kinesin-5 is a homo-tetrameric microtubule plus-end-directed motor protein that can crosslink filaments [218,219,220]. A recent study has revealed how a long four-helix bundle orients pairs of motor domains at opposite ends of this extended ~80 nm dumbbell-shaped molecule [221]. Kinesin-5 also has a non-motor microtubule binding domain at its C-terminus [222]. My lab, in collaboration with the Schmidt lab, showed that full-length *Xenopus laevis* kinesin-5 can slide apart microtubules it crosslinks [223]. Briefly, we established a “microtubule sandwich” assay in which we first immobilized axonemes (bundles of microtubules) on a glass coverslip in a flow-cell. Kinesin-5 constructs, along with stabilized microtubules and ATP, were then added, and the microtubules were imaged. Full-length kinesin-5, but not truncated dimeric constructs, captured and crosslinked microtubules from the solution and moved them relative to the surface-attached filaments. Analyses of polarity-marked filaments indicated that while kinesin-5 can slide apart antiparallel microtubules, kinesin-5-crosslinked parallel filaments do not move relative to each other. Analyses of the relative sliding of microtubules that were crosslinked at approximately right angles indicated that the motor protein walked along each filament it crosslinked. As a result, the relative sliding of two antiparallel filaments was twice the velocity of the kinesin walking along a single microtubule (Figure 5A). Subsequent studies confirmed these findings and demonstrated that kinesin-5 from other model organisms can also slide antiparallel microtubules apart [224,225].

Single molecule studies with GFP-tagged full-length *Xenopus laevis* kinesin-5 revealed that the motion of this motor protein along a single microtubule includes ATP-hydrolysis independent 1-D diffusion, in addition to its ATP-dependent directional motion [226]. We also found that crosslinking two microtubules stimulates kinesin-5’s directional motility [227]. Analyses of a homotetrameric kinesin-5 construct lacking the C-terminal non-motor microtubule binding region revealed that tetramerization of the relatively low processivity motor domains is not sufficient for relative microtubule sliding [228]. Four motor domains along with four non-motor domains in the homotetramer are needed to tune kinesin-5’s microtubule interactions for its filament sliding function. Our findings suggest a model in which kinesin-5 molecules make long (~30 s) associations with single microtubules and explore their lengths via 1-D diffusion, which together can increase the probability that kinesin-5 will crosslink another filament. If the second filament is antiparallel, kinesin-5 motility is triggered, and the motor protein walks towards each filament’s plus-end to slide them apart (Figure 5A). Interaction with a parallel filament would lead to crosslinking, but not persistent relative motion.

**Figure 6:**
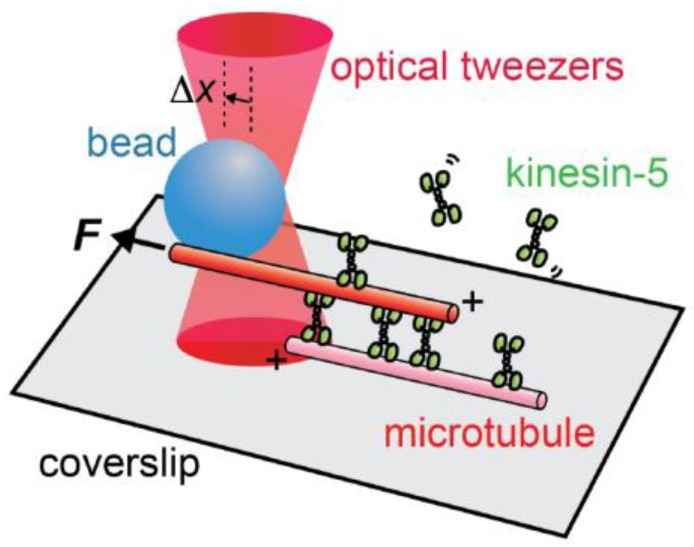
Schematic for the “mini-spindle” assay. Optical trapping and TIRF (total internal reflection fluorescence) microscopy are combined to examine forces generated by kinesin-5 sliding two microtubules apart.

Recently, we have further modified the relative filament sliding assays and now combined fluorescence imaging (TIRF (total internal reflection fluorescence) -based) with optical trapping [229] (Figure 6). This assay, which we named “mini-spindle” assay, allows imaging of microtubule and kinesin motion, as well as measurement of piconewton forces generated within this minimal structural unit of the spindle. The relative orientation and motion of the microtubules can also be directly controlled. This “mini-spindle” assay revealed that ensembles of kinesin-5 crosslinking two antiparallel microtubules can generate pushing forces that are proportional to filament overlap length.

Within the spindle, the microtubules flux poleward [6,7]. As a result, antiparallel interpolar microtubules continuously slide apart at constant relative velocities (2–3 μm/min). Our “mini-spindle” assay can mimic this relative filament motion, and also allow measurements of forces generated by kinesin-5 [229]. We find that kinesin-5 does not generate a strong pushing force when the antiparallel filament sliding velocity matches that of the motor protein’s unloaded filament sliding velocity. At slower velocities, kinesin-5 generates a pushing force to assist relative motion, and at faster microtubule sliding velocities, kinesin-5 generates a braking force. Importantly, in all these cases with moving filaments, the forces generated by kinesin-5 ensembles scale with microtubule overlap lengths.

Kinesin-5 crosslinking two parallel microtubules does not generate forces to push these filaments apart [229]. However, it does generate a substantial force to resist relative filament motion, and this force increases with relative sliding velocity. Importantly, this braking force also scales with filament overlap length.

Long-standing models for spindle assembly have predicted a force in the spindle that scales with a micrometer-scale geometric feature (e.g., filament or overlap length). Our studies with purified proteins in vitro reveal that kinesin-5 could be this activity. These findings help to interpret several observations made in the context of “whole” spindles [229]. For example, our data indicate that kinesin-5 in the spindle could help coordinate polewards flux across the dynamic micrometer-scale structure. Microtubules that slide faster relative to other filaments would experience a kinesin-5-dependent braking force, and slower filaments would experience a force that can accelerate their motion. Small microtubule “seeds” added to spindles move polewards at velocities consistent with faster dynein-dependent transport [90]. This faster polewards transport is likely possible as kinesin-5 may generate a smaller braking force for these filaments that can only achieve short overlap lengths.

### 9.2. Kinesin-12s (or hKif15/Xklp2)

These are also microtubule plus-end-directed motor proteins involved in cell division [230,231]. The addition of a dominant negative construct of the *Xenopus laevis* kinesin-12 (Xklp2) to spindle assembly reactions in egg extracts resulted in monopolar spindles [230]. Subsequent studies showed that immunodepletion of Xklp2 did not disrupt spindle assembly in the same egg extract system [206]. These findings, along with RNAi-mediated knockdown studies [232], suggest that this kinesin is not likely to be essential for spindle assembly in animal cells. However, studies in *C. elegans* have shown that kinesin-12 (KLP18) is needed for meiotic spindle formation, but not mitotic spindle formation, and may contribute to the bundling of parallel microtubules [233].

The field was relatively stuck until the Medema and Vernos laboratories re-examined kinesin-12 function in somatic cell division [234,235]. These studies devised assays based on my earlier finding that while chemical inhibition of kinesin-5 blocked spindle assembly, acute inhibitor treatments did not collapse assembled bipolar spindles in somatic cells [106]. Knockdown of kinesin-12 by RNAi in human cells revealed this motor protein was needed to maintain spindle bipolarity when kinesin-5 function was blocked using chemical inhibitors [234,235]. Knockdown of kinesin-12 alone had a modest effect on spindle assembly, but increased the fraction of monopolar spindles formed upon kinesin-5 inhibition.

Interestingly, studying somatic cell resistance to kinesin-5 inhibitors has also shed light on how kinesin-12 contributes to bipolar spindle assembly [231,236]. Ohi and co-workers have shown that kinesin-12 preferentially localizes to kinetochore microtubules, but its overexpression can also target this kinesin to interpolar microtubules [231]. This altered localization may allow kinesin-12 to function redundantly with kinesin-5 to push or keep antiparallel interpolar microtubules apart, and thereby confer resistance to kinesin-5 inhibition. Live imaging studies suggest that kinesin-12’s function in dividing cells likely involves bundling parallel microtubules. In another study, Ohi and co-workers found that resistance to kinesin-5 inhibitors can arise without over-expression of kinesin-12, but involves its function [237]. Their findings suggest that increased microtubule bundling—which can be caused by a mutation in kinesin-5—can promote kinesin-12-dependent microtubule organization and help assemble spindles in the absence of kinesin-5 motility.

Like the cell biological studies, different biochemical studies of recombinant kinesin-12 have not been entirely consistent with each other. One study from the Ohi laboratory shows that this kinesin is a homodimer with motor and non-motor microtubule binding domains [238]. Its binding to single filaments is transient, and involves the formation of an inactive “closed” conformation. By contrast, kinesin-12’s interactions with two microtubules in a bundle are long-lived. This study also showed that kinesin-12 can slide two microtubules apart [238]. However, another study from the McAinsh laboratory reports that kinesin-12 is a homotetramer that can crosslink microtubules, but cannot slide antiparallel filaments apart [239]. Both studies show that microtubule crosslinking in vitro does not require TPX2, the putative kinesin-12 spindle targeting protein. It is possible that TPX2 may promote the formation of microtubule bundles that would be the preferred substrate for kinesin-12.

Another more recent study from the McAinsh laboratory examined kinesin-12 crosslinking and sliding of dynamic microtubules in vitro [240]. The authors found that kinesin-12 can track the plus-ends of growing microtubules and suppress catastrophe when multiple motor protein molecules accumulate at filament ends. They also showed that kinesin-12 molecules can slide parallel microtubule filaments relative to each other, and suggest that this motility is achieved by differences in the velocities at which the motor walks on each filament in crosslinks (Figure 5D). Further work is needed to dissect this motor protein’s functions, and sort through some of the discrepancies in the literature.

### 9.3. Kinesin-14s (or XCTK2/HSET/Ncd)

These microtubule minus-end-directed kinesins have their motor domains at their C-terminus and a non-motor microtubule binding site at their N-terminus [241,242,243]. TIRF-based single molecule studies of the *Drosophila melanogaster* kinesin-14 (Ncd, a homodimer in solution) show that it diffuses in 1-D along single microtubules in the presence of ATP [244]. Kinesin-14 can also crosslink two microtubules in parallel or antiparallel orientations. Robust relative filament sliding (~100 nm/s) with plus-ends leading was observed only for antiparallel microtubules. Parallel filaments moved apart for a few seconds after the initial encounter, and then stopped moving. These observations can be explained by considering that when kinesin-14 molecules crosslink two filaments, the dimeric motor domains can interact with either filament (e.g., top or bottom) (Figure 5C). In the antiparallel case, motor domains walking towards the minus-end of each filament would assist each other to slide the filaments apart. In the parallel case, motor proteins walking towards the minus-ends of each filament would oppose each other in a molecular tug-of-war, and the filaments would not move relative to each other. Analyses of the *S. pombe* kinesin-14 (klp2) revealed a similar activity [245], suggesting that potentially all kinesin-14s slide antiparallel filaments apart and generate static crosslinks between parallel filaments.

These elegant in vitro studies indicate that kinesin-14s could oppose the relative filament sliding of antiparallel microtubules driven by kinesin-5, as these motor proteins would push filaments apart in opposite directions. It is noteworthy that while kinesin-5 molecules would be stationary between two filaments that slide apart, kinesin-14 molecules would translocate with the moving filaments, diffusing and switching orientations between the two crosslinked filaments.

### 9.4. Cytoplasmic Dynein

This microtubule-based motor protein is a member of the AAA+ (ATPases associated with diverse cellular activities) family [246]. It functions as an ~1.2 MDa multi-protein complex with two heavy chains, each of which contains a motor domain at the C-terminus and a “tail” domain that mediates interactions with accessory factors at its N-terminus [247,248,249]. Cytoplasmic dynein is the major microtubule minus-end-directed motor protein that transports a wide range of cargoes (e.g., organelles, proteins, and mRNA) in eukaryotic cells. There have been several advances in our understanding of cytoplasmic dynein’s structure, biochemistry, and motility [247,248,249]. Here I highlight dynein’s function in spindle organization, focusing on its microtubule sliding and sorting functions. This activity is needed to organize the prophase microtubule array [115], assemble spindle poles [250,251,252], and to generate forces that counteract the activities of kinesin-5 and kinesin-12 [234,235,253,254,255]. These functions have been linked to the transport of other microtubule-associated proteins (e.g., NuMA [250]), and to the relative sliding of microtubules [14,251].

Early biochemical studies demonstrated the crosslinking and bundling of microtubules by dynein in vitro [256,257]. A recent study by Tannenbaum and co-workers has shown that cytoplasmic dynein can also slide antiparallel microtubules apart [236]. The use of well-characterized truncated constructs revealed that this relative microtubule sliding activity is independent of “tail”-domain-mediated interactions with accessory proteins. Single molecule studies revealed that dynein, which walks towards the minus-ends of single filaments, has a much more complex behavior when interacting with two antiparallel microtubules. Dynein molecules can pause for seconds before making directional runs along either filament they crosslink. These data, together with additional findings, suggest a model in which dynein can crosslink and slide microtubules in a manner distinct from kinesin-5 and kinesin-14. Each AAA domain in the dimeric molecule can bind a different filament, and walking on each filament would lead to relative motion, pulling microtubule minus-ends together (Figure 5B).

### 9.5. In Vitro Studies of Motor Proteins Generating Opposing Forces

It is tempting to speculate that motor proteins with opposing activities should be able to establish stable antiparallel microtubule overlap—a recurring filament configuration within the metaphase spindle. However, theoretical work has suggested that motor protein mixtures would lead to unstable states [258,259,260]. Experiments are consistent with the theory. Directional instability (i.e., with frequent back and forth motion of the microtubules) has been observed when mixtures of dynein and kinesin-1 [261], or kinesin-5 and kinesin-14, are immobilized on surfaces [222]. Another study that examined the relative sliding of two antiparallel microtubules by mixtures of kinesin-5 and kinesin-14 also indicated that static overlap cannot be achieved [224]. In fact, efforts to balance the motor proteins’ activities resulted in directional instability, with a broad distribution of instantaneous velocities [224]. Interestingly, this study also reveals that a much smaller number of kinesin-5 molecules can compete against a large number of kinesin-14 molecules crosslinking antiparallel microtubules. Importantly, this study shows that stable antiparallel microtubule overlap can be achieved by incorporating an engineered microtubule crosslinking protein along with the motor proteins. There are several filament crosslinking proteins that are known to be involved in cell division, and it is likely that these proteins may contribute to stabilizing filament overlap. Constant antiparallel microtubule overlap in animal spindles must be maintained in the presence of persistent motion due to poleward flux [6], adding another layer of complexity to the efforts in reconstituting spindle assembly with purified proteins.

## 10. Outlook

As outlined in this review, there have been several major advances in our understanding of how the cell division apparatus assembles. We are now poised to unravel the basic biochemical principles underlying many of the processes, such as microtubule nucleation and force generation, required for successful cell division. New cell-based experiments will help test specific predictions from biochemical studies. The multiple regulatory inputs controlling each of these essential processes will have to be carefully teased apart. I believe that now is a very exciting time to study cell division, as it provides a unique opportunity to establish the basic principles for how micrometer-scale cellular structures can be assembled by nanometer-sized proteins, and also explore how mechanical and biochemical inputs intersect to control distinct protein outputs. Powerful new methodologies (e.g., to image cell division in different tissues in vivo) and tools (e.g., cell-permeable chemical inhibitors of key proteins) will likely be developed and used to answer these questions.
